# Osteonecrosis of Femoral Head Occurred after Stent Placement of Femoral Artery

**DOI:** 10.1155/2014/727949

**Published:** 2014-08-17

**Authors:** Akiyoshi Shimatani, Fumiaki Inori, Taku Yoshida, Masahiko Tohyama, Sadahiko Konishi, Hirotsugu Ohashi

**Affiliations:** ^1^Department of Orthopedic Surgery, Osaka General Hospital of West Japan Railway Company, Osaka 545-0053, Japan; ^2^Department of Orthopedic Surgery, Osaka Saiseikai Nakatsu Hospital, Osaka 530-0012, Japan

## Abstract

We present a case of osteonecrosis of femoral head (ONFH) that occurred after stent angiography of femoral artery for the treatment of arteriosclerosis obliterans (ASO) of left inferior limb in a 76-year-old woman. No case of late collapse of femoral head as a complication of endovascular procedure such as stent placement has been previously documented. We considered that ONFH occurred after detaining stent at a junction of left deep femoral artery for the treatment of the ischemia of left lateral and medial femoral circumflex artery.

## 1. Introduction

ASO is a disease that significantly impacts patients' quality of life because of leg pain, ulceration, and gangrene. We report a case of the ONFH that occurred after the stent placement for the treatment of the ischemia of lateral and medial femoral circumflex artery.

## 2. Case Report

**Figure 1:**
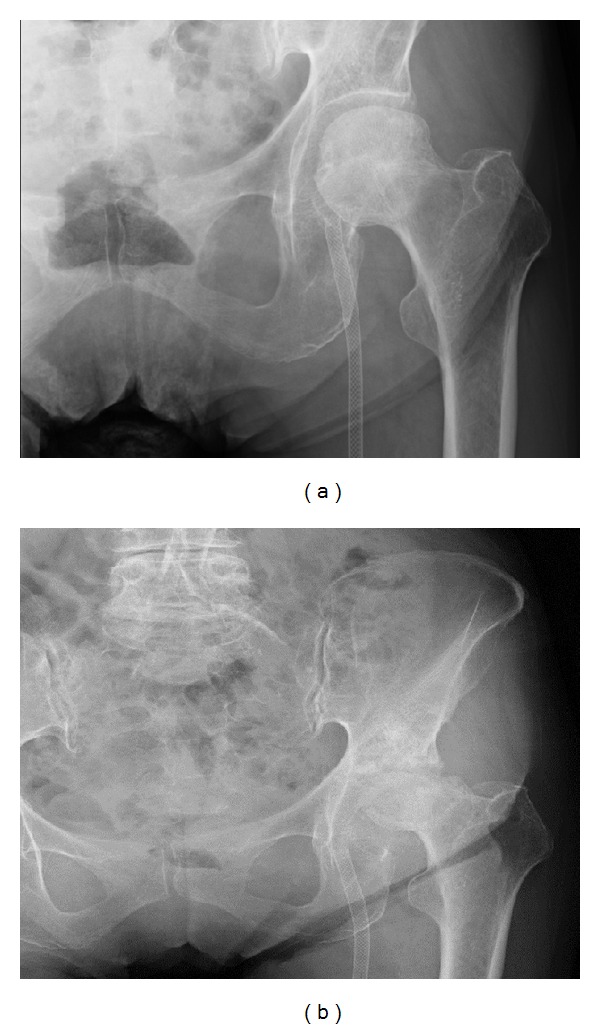
Plain radiography. (a) Just after the stent placement in another hospital. Stent is detained in left femoral artery. Left femoral head was not collapsed. (b) At the time of admission to our hospital. Left femoral head was collapsed and joint space disappeared.

A 76-year-old woman complained of left inferior limb pain and intermittent claudication. She was diagnosed with ASO in another clinic about 10 years ago. While the prolonged conservative medical treatment was undergone, the condition got worse. Then, she consulted a vascular surgeon in another hospital and the angiographic examination was undergone. The complete obstruction of left femoral artery was observed. Percutaneous transluminal angiography (PTA) was performed and stent was detained at the left femoral artery (Figure 1(a)). Revascularization was achieved and she could walk about 300 meters postoperatively. However, the left inferior limb pain still remained. Since the limb pain got worse, she was not able to walk five months after detaining stent. She was diagnosed with rapidly destructive coxarthrosis by plain radiography in an orthopaedic clinic and admitted to our hospital for the purpose of total hip arthroplasty (THA). She had no history of a large quantity of alcohol or steroid drug intake.

**Figure 2:**
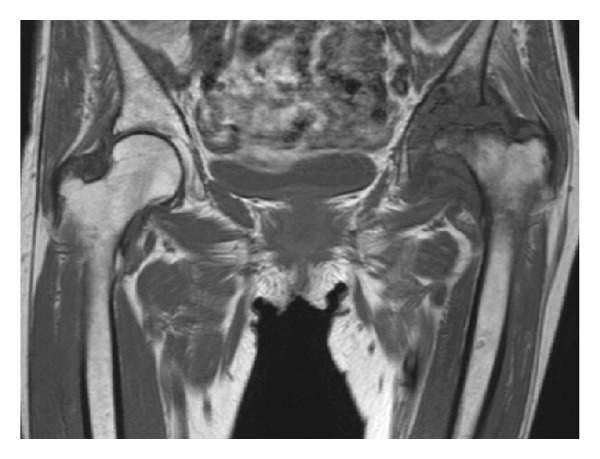
MRI T1WI at the time of admission to our hospital. Femoral head was completely collapsed and low band was observed in the upper edge of the femoral head.

**Figure 3:**
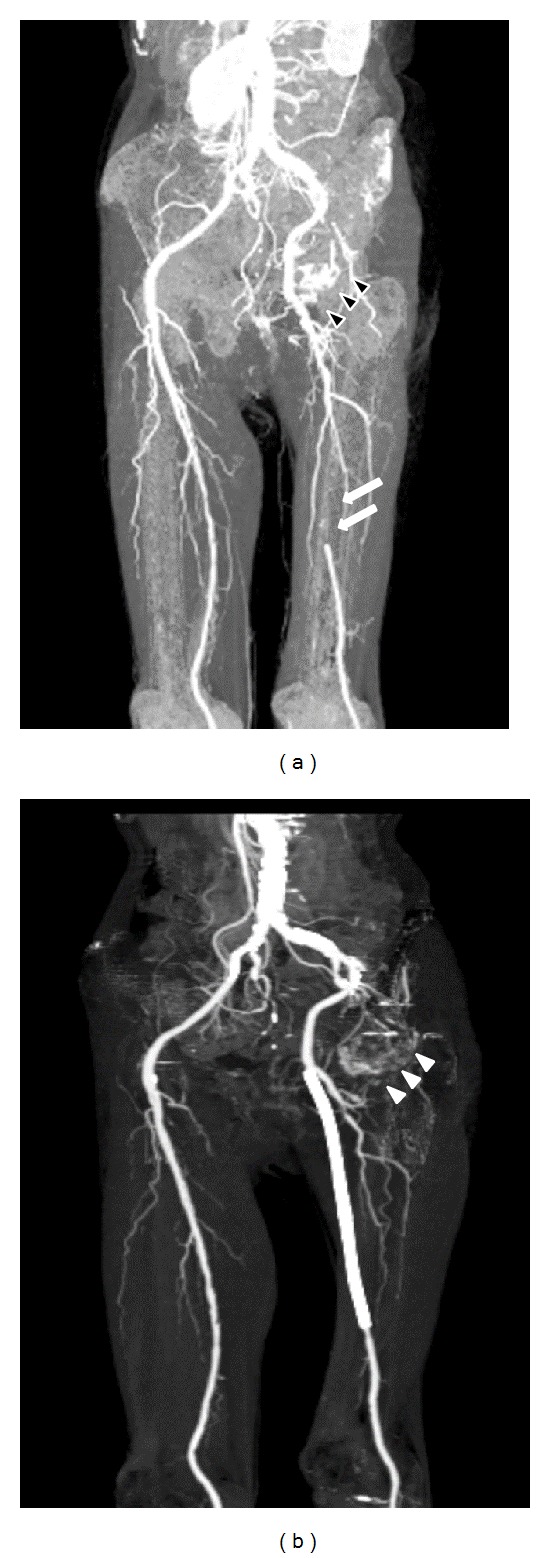
(a) Angiography before PTA taken at another hospital. Complete obstruction of left femoral artery before PTA (white arrow) was observed. Blood stream for left femoral head was alive (black arrow head). (b) Angiography taken just before THA at our hospital. Revascularization of blood vessels (white arrow head) was observed around left hip joint after PTA.

**Figure 4:**
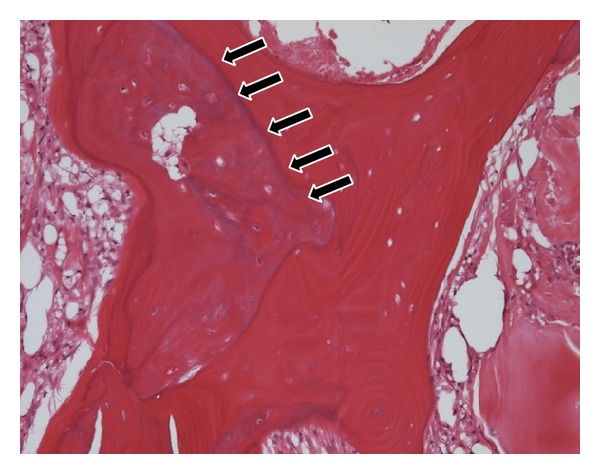
Microscopic findings in high magnification field (×100, HE stain). New bone formation is observed adjacent to necrotic trabecular bone characterized to empty lacuna (black arrow).

Plain radiography showed the stent which is detained in left femoral artery, collapse of left femoral head, and disappearance of joint space (Figure 1(b)). MRI T1WI showed bone marrow edema, collapse of left femoral head with appearance of low band, and pooling of joint fluid (Figure 2). Terminal stage of coxarthrosis was diagnosed from these findings. Angiography before PTA showed complete obstruction of left femoral artery; however bloodstream for left femoral head could be identified (Figure 3(a)). Angiography taken just before THA at our hospital showed increasing of blood vessel around left hip joint (Figure 3(b)). These findings suggested compensation of the ischemia. Six months after detaining stent, THA was performed with noncemented cup and cemented stem. Macroscopic findings of the resected femoral head showed severe osteoarthritic change, such as flattening of the upper part of femoral head and osteophyte formation. From microscopic findings in high magnification field (×100), empty lacuna and addition of newly formed bones adjacent to those necrotic bone areas were observed (Figure 4). These histological findings indicated that bone necrosis occurred firstly and osteoarthritic change progressed secondarily.

## 3. Discussion

ONFH is a pathologic process that results from interruption of blood supply to the bone. Femoral head ischemia results in the death of marrow and osteocytes and usually results in the collapse of the necrotic segment. Focusing on nontraumatic osteonecrosis, there are various possible theories, such as fat-emboli theory [1], increased marrow internal pressure theory [2, 3], malfunction of blood coagulation theory [4, 5], vascular lesion theory, and microfracture theory. However, the detailed mechanism of the ischemia in the femoral head was still not clear. The blood flow of femoral head was supplied mainly from the lateral and medial femoral circumflex artery and the artery of ligamentum capitis femoris [6–8]. In this case, the stent is detained at a junction of left deep femoral artery. This detained stent might occlude the left lateral and medial femoral circumflex artery, resulting in osteonecrosis of femoral head.

Kubo et al. reported that the collapse of femoral head progressed in an average of 3 years and 4 months (8 months to 7 years) after steroid use for renal transplanted patients [9]. Ohzono et al. reported that the collapse of femoral head developed within one year in 20% of nontraumatic osteonecrosis cases [10]. Compared with these reports, collapse of femoral head in this case progressed in a short period, about 5 months. In several case reports osteonecrosis of femoral head occurred after vascular injury; relatively short period to the progress of symptom has been described. In case ONFH occurred following embolization of the right medial femoral circumflex artery after a failed prior internal iliac artery ligation to control benign pelvic haemorrhage, it took about 2 years for the progress of symptoms [11]. In case of osteonecrosis of femoral head associated with disruption of splenic artery, it took about 2 months [12]. Considering that in this case ONFH occurred due to the occlusion of the left lateral and medial femoral circumflex artery, the short period to the progress of symptom was compatible with these reported cases.

In summary, we experienced a case of ONFH after stent placement at a junction of left deep femoral artery for the treatment of the ischemia of left lateral and medial femoral circumflex artery. There are several reports of ONFH after the medical treatment such as embolization [11, 12]; however there is no report of ONFH after revascularization by stent as far as we examined.

## References

[1] Jones JP (1993). Fat embolism, intravascular coagulation, and osteonecrosis. *Clinical Orthopaedics and Related Research*.

[2] Wang TY, Avlonitis EG, Relkin R (1988). Systemic necrotizing vasculitis causing bone necrosis. *The American Journal of Medicine*.

[3] Hofmann S, Engel A, Neuhold A, Leder K, Kramer J, Plenk H (1993). Bone-marrow oedema syndrome and transient osteoporosis of the hip. An MRI-controlled study of treatment by core decompression. *Journal of Bone and Joint Surgery B*.

[4] Glueck CJ, Freiberg R, Tracy T, Stroop D, Wang P (1997). Thrombophilia and hypofibrinolysis: pathophysiologies of osteonecrosis. *Clinical Orthopaedics and Related Research*.

[5] Kubo T, Tsuji H, Yamamoto T, Nakahara H, Nakagawa M, Hirasawa Y (2000). Antithrombin III deficiency in a patient with multifocal osteonecrosis. *Clinical Orthopaedics and Related Research*.

[6] Trueta J (1957). The normal vascular anatomy of the human femoral head during growth. *The Journal of Bone and Joint Surgery*.

[7] Sevitt S, Thorrtpson RG (1965). The distribution and anastomosis of arteries supplying the head and neck of the femur. *The Journal of Bone and Joint Surgery*.

[8] Xiao J, Yang X, Xiao X (2012). DSA observation of hemodynamic response of femoral head with femoral neck fracture during traction: a pilot study. *Journal of Orthopaedic Trauma*.

[9] Kubo T, Yoshimura N, Oka T (1998). Long-term X-ray follow-up osteonecrosis of the femoral head after renal transplantation. *Transplantation Proceedings*.

[10] Ohzono K, Saito M, Takaoka K (1991). Natural history of nontraumatic avascular necrosis of the femoral head. *Journal of Bone and Joint Surgery B*.

[11] Obaro RO, Sniderman KW (1995). Avascular necrosis of the femoral head as a complication of complex embolization for severe pelvic haemorrhage. *British Journal of Radiology*.

[12] Puylaert D, Nuyts R, Ramael M, Verstreken J (1995). Rapidly progressive destruction of the hip. Case report and review of the literature.. *Acta orthopaedica Belgica*.

